# Radiations and male fertility

**DOI:** 10.1186/s12958-018-0431-1

**Published:** 2018-12-09

**Authors:** Kavindra Kumar Kesari, Ashok Agarwal, Ralf Henkel

**Affiliations:** 1 Department of Applied Physics, Aalto University, Espoo, Finland; 2 American Center for Reproductive Medicine, Cleveland Clinic, Mail Code X-11, 10681 Carnegie Avenue, Cleveland, OH 44195 USA; 3 Department of Medical Bioscience, University of the Western Cape, Robert Sobukwe Road, Bellville, 7535 South Africa

## Abstract

During recent years, an increasing percentage of male infertility has to be attributed to an array of environmental, health and lifestyle factors. Male infertility is likely to be affected by the intense exposure to heat and extreme exposure to pesticides, radiations, radioactivity and other hazardous substances. We are surrounded by several types of ionizing and non-ionizing radiations and both have recognized causative effects on spermatogenesis. Since it is impossible to cover all types of radiation sources and their biological effects under a single title, this review is focusing on radiation deriving from cell phones, laptops, Wi-Fi and microwave ovens, as these are the most common sources of non-ionizing radiations, which may contribute to the cause of infertility by exploring the effect of exposure to radiofrequency radiations on the male fertility pattern. From currently available studies it is clear that radiofrequency electromagnetic fields (RF-EMF) have deleterious effects on sperm parameters (like sperm count, morphology, motility), affects the role of kinases in cellular metabolism and the endocrine system, and produces genotoxicity, genomic instability and oxidative stress. This is followed with protective measures for these radiations and future recommendations. The study concludes that the RF-EMF may induce oxidative stress with an increased level of reactive oxygen species, which may lead to infertility. This has been concluded based on available evidences from in vitro and in vivo studies suggesting that RF-EMF exposure negatively affects sperm quality.

## Introduction: History and sources of microwaves

Radiation can be characterized into ionizing and non-ionizing radiations, of which the latter is differentiated in two forms: 1) extremely low frequency (ELF) or power line (60 Hz) electromagnetic fields (EMFs), and 2) radio frequency (RF) EMFs - which are produced by wireless radio waves/microwaves products.

The biological effects of microwave radiations effectively begin with the development of radar early during World War II. No harmful effects of microwaves were detected prior to this time and are also not in the list of a general environmental problems. Prausnitz and Susskind were the first who reported the effects of microwave radiation on the testicular organ in 1962 [1]. Since early 1962, many man-made devices are now in use and the most common source for microwaves are transmission lines (50–60 Hz), computer monitors (60–90 Hz), AM radio transmissions (530–1600 KHz), FM radio transmissions (88–108 MHz), television transmissions (50–700 MHz), hand phones (850 MHz-2.4 GHz), microwave ovens (2.45 GHz), laptops and Wi-Fi (2.4 GHz).

**Fig. 1:**
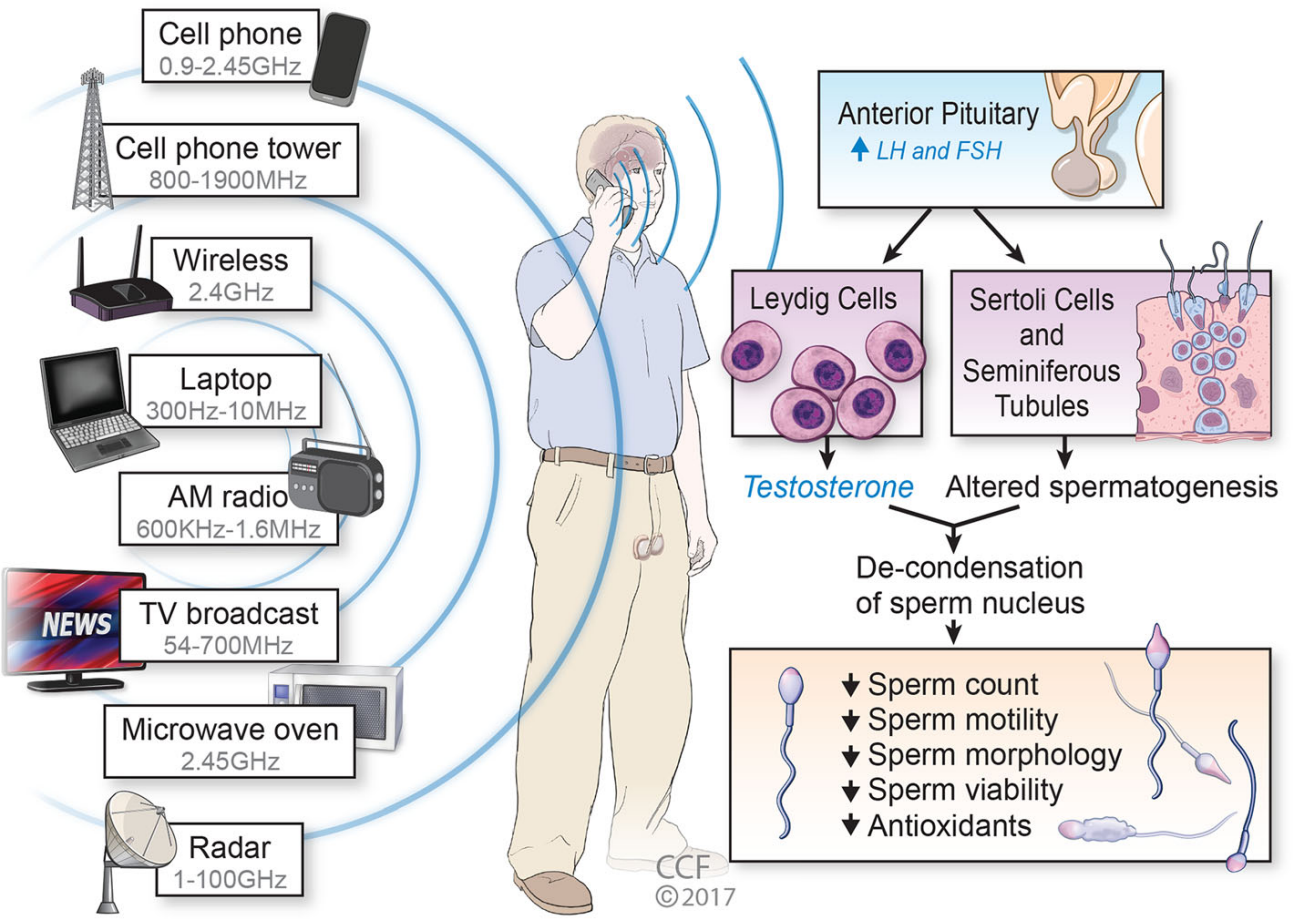
Diagrammatic representation of various source of RF EMF exposure effect on brain and testicular organ and deleterious outcome

The frequencies in the range of 100 kHz to 300 GHz refer to RF and represent only a part of the electro-magnetic spectrum. Figure 1 shows the sources of radiofrequency electromagnetic field (RF-EMF) exposure affecting sperm parameters. In the list of new technologies, intermediate frequency (IF) has been listed as newest source of exposure to electro-magnetic fields. This frequency range falls between the low frequency (low frequency- 0.1 Hz–1 kHz) and the radio frequency (RF) (10 MHz–300 GHz). Major sources of this range are airport security scanners and anti-theft devices operated at the exits of shops.

On the other hand, radiations such as X-rays, γ-rays and α-particles are forms of ionizing radiation [2]*.* Ionizing radiation is much more dangerous than non-ionizing radiations. Significant sources of ionizing γ-rays include natural sources such as the decay of uranium in the earth, cosmic rays, the sun and radon gas, while artificial or manmade sources include radioactive waste, X-rays from medical procedures etc.

Radiation induced cancer is triggered by chromosomal damage or genomic instability [3]. An increase in chromosomal abnormalities may be a result of exposure to radiation, which was first reported by Martin et al. [4]. The most radiosensitive organ reported is the male testis with the germinal epithelium including the spermatogonia which are more sensitive to radiation exposure than other cells [5, 6].

The effects of IR on reproduction are of growing concern as the number of people exposed to radiation via medical procedures and environmental exposures is significantly increasing. Data reviewed by Yousif et al. [7] obtained from 31 studies report an association between occupational exposures to IR and either the incidence of or mortality from testicular cancer. Similar data were obtained for non-ionizing radiation from 9 studies. Since radiations have a broad range of wavelengths, it is impossible to cover all the existing ionizing and non-ionizing radiations in a single article. Further, the available data on ionizing radiation clearly indicate its role in the development of cancers, such as testicular cancer. In contrast, much less clear information is available on non-ionizing radiation. Therefore, the focus of our review is more to explore the effects of non-ionizing radiation such as RF-EMF on male fertility. This includes frequencies used for cell phones, laptops, computers, microwave ovens and some other higher frequency range; this includes the RF-EMF-induced biological effects and potential mechanisms on the male reproductive system.

The networking of RF-EMF-assisted devices like cell phones, Wi-Fi, microwave ovens, and laptops is increasing drastically and its association with male infertility has been reported [8–11]. Based on sufficient evidence, it has now been increasingly realized that RF-EMF radiation is pervading the environment and has therefore been mentioned under the terms “electro-pollution” or “electro-smog” in the list of other environmental pollutants (air, water, soil, and noise pollution) [12].

The International Agency for Research on Cancer [13, 14] classified RF in group 2B as ‘possibly carcinogenic’ to humans. The guidelines on the specific absorption rate (SAR) of mobile phones are legally limited to 2.0 W/kg by the International Commission on Non-Ionizing Radiation Protection reported [15], but still the SAR level varies from country to country. SAR is a standard unit or rate at which RF-EMF energy is imparted to an element or mass to measure the penetration of energy within human tissues.

The amount of SAR absorbed by human tissue depends on many factors such as the frequency, intensity, polarization and duration of exposure [16] and most importantly the position of devices while used. A higher radiation absorption rate could be observed while talking on phone, keeping phone near head or in pants pocket, using laptop computer on lap connected with Wi-Fi and frequently use of microwave ovens. Agarwal et al. suggested that using mobile phones adversely affects the quality of semen by decreasing the sperm count, motility, viability and morphology, which might contribute to male infertility [17]. Consequently, Desai et al. concluded that RF-EMF exposure might induce DNA damage due to increased oxidative stress, which may accelerate spermatozoal cell death and promote testicular carcinogenesis [18]. Many animal studies on the use of mobile phones are linked to a reduction in sperm count [9] and motility [19], suggesting an impairment of male fertility. Similarly, in humans, Agarwal et al. reported that the continuous use of mobile phones is associated with decreased motility, sperm concentration, morphology and viability [20]. The most significant studies on the effect of RF-EMF emitted from different sources (cell phones, microwave ovens, laptops, and Wi-Fi devices) on animal and human fertility pattern are summarized in Table 1 [21–37].

**Table 1:** Studies on Reproduction: In vitro & In vivo

Subject/ species	Exposure Parameters	Findings	References
Male swiss albino mice, n = 8	902.4 MHz 4 h/ 8 h/ day for 35 days; SAR 0.0516 W/kg	Significant increase in abnormal cells, spermatogonia and decreased spermatids. Significant histological changes in seminiferous tubules. Significant increase in DNA damage of both 4 & 8 h exposure.	Pandey et al. 2017 [21].
Sprague Dawley male rats, *n* = 8	900 MHz mobile phone frequency; 1 h/ day for 30 days; SAR 0.025 W/kg	Significant increase in apoptosis and changes in the levels of SOD, GPx, CAT, LPO. Cincludes that 900 MHz could alter histology, the oxidative status and apoptosis induction in testes.	Odaci and Ozyilmaz 2016 [22]
Human spermatozoa (in vitro), *n* = 26	850 MHz continuous for 1 h; SAR 1.46 W/kg	In group 1 of normal sperm, the gene and protein expression of clusterin and DNA fragmentation were increased significantly in EMF exposed sperm. Concluded detrimental effect of mobile phone on sperm parameters.	Zalata et al. 2015 [23]
Human spermatozoa (in vitro), *n* = 32	900–1800 MHz; intermittent every 10 min for 5 h;	Significantly increase in DNA fragmentation and non-progressive motility and reduction in progressive motility in exposed sperm.	Gorpinchenko et al. 2014 [24]
Male Wistar rats, *n* = 6	2.45 GHz/0.14 W/Kg (2 h a day for 45 days)	A significant increase in DNA SB, protein carbonyl content, ROS, XO, MDA apoptosis and significant decrease in testosterone, LDH-X were observed in microwave exposed group. A treatment with melatonin prevent oxidative damage in all above parameters.	Meena et al. 2014 [25].
Male Wistar rats, n = 6	1910.5 MHz/ 1.34 W/kg 60 days, two hours each day (6 days a week)	Significant decrease in sperm count, seminiferous diameter, testicular weight and increase in DNA single strand break and MDA level.	Kumar et al. 2014 [26]
Male Male Wistar rats, n = 6 in each group	GSM 900 MHz/ 0.9 W/Kg (2 h/day for 45 days	Decrease sperm count, increased apoptosis, micronuclei and ROS. Affect the level of antioxidant enzymes and testosterone level. Morphological changes also observed under TEM.	Kesari et al. 2011, Kesari and Behari 2012 [27, 28]
Male Wistar rats, n = 3 each group	2.45GHz/ 0.014 W/Kg (2 h/day for 60 days). PEMF 100 Hz	Decreased melatonin, testosterone and increased creatine kinase, capases significantly in exposed group. PEMF showed therapeutic impact against microwave exposure.	Kumar et al. 2011 [29]
Male Wistar Rats, n = 6 each group	10GHz/ flux density 0.21 mW/cm^2^/ SAR: 0.014 W/kg/ Continuous 2 h/day for 45 days	Significant increase in ROS level, apoptotic cells and decrease in percentage of G_2_ phase /mitosis phase of cell cycle and histone kinase enzyme activity.	Kumar et al. 2011 [30]
Male Wistar rats, n = 6	2.45GHz/ 0.11 W/Kg (2 h/day for 35 days)	Significant decrease in sperm count, changes in antioxidant enzyme (SOD, GPx, CAT) and DNA fragmentation exceed to cell apoptosis.	Kesari and Behari 2010 [31]
Male Wistar rats, n = 6	RF-EMR 900/ 0.9 W/kg (2 h/ day for 35 days)	Statistically significant reduction in Protein Kinase C activity, sperm count and increased apoptotic sperm cells.	Kesari et al. 2010 [32]
Male Albino Wistar rat	900 MHz GSM (60 min/day for 3 months)	Long term mobile phone radiation exposure leads to reduction in serum testosterone level	Meo et al. 2010 [33]
Human semen	RF-EMR 850 MHz/ 1.46 W/kg. (for 60 min)	Motility & viability significantly decreased, increased in ROS level, decreased in ROS-TAC score	Agarwal et al. (2009) [34]
Male Albino Wistar rat	GSM 0.9 & 1.8 GHz/ SAR-? (1 h/day for 28 days)	Reduced % of motile sperm. Increase LPx, GSH content of testis and epididymis.	Mailankot et al. *2009* [35]
Human Spermatozoa	71.8 GHz/ 0.4–27.5 W/Kg (exposure time 16 h).	Both [power density and frequency range enhance mitochondrial ROS in human spermatozoa leads to decrease in motility and viability and cause DNA fragmentation	De Iuliis et al. 2009 [36]
Sprague Dawley rats	RF-EMR 1.9 Hz @ distance of 1 cm for 6 h/day × 18 weeks	Significant decrease in sperm motility also majority of sperm cells in the exposure group were dead, where as in the control group the majority were alive with constant, active motility	Yan et al. 2007 [37]

The literature shows that studies investigating the deleterious effects of cell phone and microwave exposure on male reproductive organs are mainly concentrating on sperm parameters [9, 25, 38]. However, till date, no possible mechanisms on how RF-EMF radiation interacts with the male reproductive organs and thereby affect the fertility pattern are known. Some of the concerns are listed and discussed in detail by introducing 1) biophysics of RF-EMF radiation, 2) effect of RF-EMF on sperm parameters 3) role of kinases in cellular metabolism 4) genotoxic effect of EMF leading to genomic instability 5) RF-induced oxidative stress 6) RF-EMF effect on reproductive endocrine system, and 7) protective measures for these radiations and future recommendations.

### Biophysical parameters of RF-EMF

The biophysical parameters describe the physical and biological factors, which determine cellular radio-sensitivity of RF-EMF exposure by measuring the absorption rate of the radiation. In theory, the EMF must penetrate the exposed biological system and induce internal EMFs to cause a biological response. On the other hand, the penetration depth or RF radiation absorption depends on incident field parameters (like intensity, power density), zone of exposure, shape, geometry, and orientation of the object; and configuration of the radiation, e.g., how close is the object from the RFR source? [39]. These parameters directly or indirectly participate in free radical formation by increasing ROS levels, which have been found to be a factor for DNA damage. Kumar et al. have reported sperm DNA damage after 3G mobile phone exposures [26].

DNA damage is one of the serious concerns in respect to infertility or testicular cancer. The question, however, is how such a low frequency RF radiation may cause DNA damage? This question is not easy to answer, but it is assumed that a RF electro-magnetic field is classified as non-ionizing radiation because the photons do not have sufficient energy to break chemical bonds or directly ionize biological molecules [39]. Therefore, it is generally accepted that the EMF energy is not enough to damage DNA directly, thus indirect mechanisms, such as the free radical hypothesis, have been proposed to explain EMF-induced DNA damage [40–42]. Cell phones and its transmission towers, are both equally responsible for health effects, as cell phones emit radiations to nearby relay base stations or antennas. Our bodies act as antennas that absorb the radiation and convert it into alternating eddy currents [43]. Cell phone radiation is generated in the transmitter, and is emitted through the antenna in the form of radio waves [16, 39, 44]. The impact of this RF-EMF on the human body is measured via a standardized unit called the SAR. The rate of energy absorbed by or deposited per unit mass per unit time is the SAR and E-filed can be calculated by-

$$ SAR\left(W/ Kg\right)=\sigma {E}^2/\rho $$

Where sigma (σ) is the conductivity of the liquid and rho (*ρ*) is the density of liquid. The measured E-field values and SAR distribution are 1 g and 10 g mass averaged SAR values.

When a biological body or tissue is exposed to RF-EMF, the RF energy is scattered and attenuated as it penetrates body tissues. Energy absorption is largely a function of the radiation frequency and the composition of the exposed tissue. The problem of physics in respect to EMF exposure is of penetration depth. The higher absorption rate of radiations emitting from cell phone is more absorbed inside the tissue while making a cell phone call or using electro-magnetic devices.

Testicles are very sensitive to these radiations because of the development and maturation processes of sperm taking place in the testicles. It is also well established that the developing phase of the brain and the testicles are very sensitive to radiation, which may cause severe damages in the form of genotoxic effects [9, 25, 26, 45]. Several studies suggest that microwave radiations are potentially strong enough to penetrate the brain cranium, and nearly 40% of these can reach deeper into the brain [46, 47]; penetration depths of 4–5 cm are assumed [48, 49]. The same applies to the testes.

During testicular developmental stages, the penetration depth is not the sole factor, but also i) exposure time; ii) duration of exposure (i.e. number of exposure days); iii) the greater number of undeveloped cells exposed to microwaves; and iv) the water content of the organ (the greater the amount of water in an organ, the greater will be the effect of the microwave radiation). Several studies also reported that EMF-induced morphological changes are also depending on the type, dose, mode and duration of the EMF-exposure [50–54]. Therefore, it is imperative to explore biophysical parameters related to RF-EMF exposure and causative factors, first.

### The effect of RF-EMF exposure on sperm parameters

In light of reports indicating that in 2005 7.4% of couples in the United States were infertile [55], and that this number is predicted to increase as high as 15%, particularly in industrialized countries [56], one can link the increasing usage of RF devices such as cell phones or Wi-Fi, with R F-EMF induced sperm damages as this is closely related to infertility. Although, there are numerous other factors such as sperm quality, sperm count, motility and morphology impair with increasing age, and lifestyle factors for example alcohol consumption, cigarette smoking that may affect fertility pattern in both male and female, frequent use of cell phone or EMF devices contribute markedly to this poor semen quality (Figure 1).

Apart from this, cell phone usage has been linked to decreases in progressive motile sperm count [20] motility [20] and viability [20, 34], as well as to increases in ROS [29] and abnormal sperm morphology. Recent evidence also shows that Wi-Fi from laptops negatively affects sperm quality [8]. EMF is also responsible for the decrease in fertilization rate [57], spermatogenic cell numbers and trigger apoptosis [58, 59], reduced sperm quality [60], hormonal changes in the testis [20, 61], and may give rise to fetal loss and developmental impairments in the embryonic period [45, 62] (Table 2) [9, 20, 25, 26, 28–32, 34, 38, 63].

**Table 2:** Studies reporting from our group on sperm parameters after RF EMF exposure to male subject. The studies indicated by arrow in table are either significant increase or decrease in given endpoints

Reference	RF-EMF	Sperm Count	Sperm morphology	Sperm Motility	Sperm cell cycle	Testosterone	ROS	Comment/ Exposure
Kumar et al. [63]	Microwaves 10 GHz		↓	↓		↓		Male Wistar rat 2 h/d/45d
Kesari et al. [9]	Mobile phone 900 MHz	↓					↑	Male Wistar rat 2 h/d/35d
Agarwal et al. [20]	RF-EMR 850 MHz	↓	↓	↓				Human semen
Meena et al. [25]	Microwaves 2.45 GHz	↓	↓			↓	↑	Male Wistar rat 2 h/d/45d
Kumar et al. [26]	3G mobile phone	↓		↓				Male Wistar rat 2 h/d/60d
Kesari & Behari [28]	Mobile phone 900 MHz		↓	↓		↓	↑	Wistar rat 2 h/d/45d
Kumar et al. [29]	Microwaves 2.45 GHz					↓		Male Wistar rat 2 h/d/60d
Kumar et al. [30]	Microwaves 10 GHz				↓		↑	Male Wistar rat, 2 h/d/45d
Kesari and Behari 2010a,b [31, 32]	Microwaves 2.45 & 50 GHz	↓	↓		↓			Male Wistar rat 2 h/d/45d
Agarwal et al. [34]	RF-EMR 850 MHz			↓			↑	Human semen
Kesari et al. [38]	Mobile phone 900 MHz	↓			↓		↑	Male Wistar rat, 2 h/d/35d

#### Sperm count

Radio-frequency electro-magnetic field exposure from cell phones or other sources of microwaves adversely affect male fertilizing potential of spermatozoa [29]. There are several techniques available for the measurement of sperm count like, hemocytometer, flowcytometry and cell counter. Using flowcytometry, Kesari et al. showed a significantly (*P* < 0.0001) decreased percent of sperm count (61.33 ± 3.68% vs. 31.14 ± 13.6%) and an increased percentage of apoptotic cells (5.93 ± 1.64% vs. 13.15 ± 1.26%) after cell phone exposure (2 h/day for 35 days) in an animal study [9]. In addition to cell phone radiation, the exposure of male Wistar rats to Wi-Fi connected laptop computers (EMF, 1.15 micro Tesla, μT) for 7 h/day for 1 week also reduced sperm count and motility [64]. Other studies have also linked RF-EMF [34, 37, 61, 65, 66] or cell phone radiation [67–69] to deleterious effects on the testes. Such radiation exposure may create a state of oxidative stress and stimulates free radical generation by the sperm mitochondria [67].

#### Sperm motility and morphology

There is also a list of studies indicating the negative influence of RF-EMF on sperm motility and morphology. Several authors found that carrying GSM phones in the trouser pocket or on the belt decreased rapid progressive motility of sperm [70, 71]. Kesari and Behari demonstrated that males who use mobile phones exhibit increased rates of abnormal sperm morphology [28]. Several groups showed that men using mobile phones have decreased sperm concentration, motility, normal morphology, and viability [16, 28, 37, 72, 73]. Further, Luo et al. [74] showed that RF-EMF exposure is directly affecting the testes by causing a significant decrease in the diameter and weight of the seminiferous tubules as well as the mean height of the germinal epithelium and pathological and physiological changes in testicular tissues, respectively, thus, giving evidence for the growing concerns of increasing incidences of infertility [17, 26].

The link between the exposure to RF-EMF and testicular pathologies and decreasing sperm quality is most probably oxidative stress by increasing levels of free radicals or superoxide anion as a decrease in sperm motility and viability is triggered by increasing concentrations of superoxide anion (^•^O_2_^−^) [34]. Free radicals oxidize membrane phospholipids extracellularly, thus causing decreased viability and reduced membrane fluidity with impaired motility.

#### Role of kinases in sperm cell cycle and apoptosis

Apoptosis plays an important role in adjusting the appropriate number of proliferating germ cells associated with the surrounding Sertoli cells during spermatogenesis [75, 76]. Apoptosis or programmed cell death in the tissues of an organism is an important and inevitable event in the remodeling of tissues during development and spermatogenesis [77]. Cell cycle analysis by flow cytometer has confirmed these results because EMF exposure induces the appearance of a sub-G1 apoptotic peak, which is characteristic of DNA fragmentation in spermatozoa [30]. Cell phone radiation exposure showed a significant decrease in G_0_–G_1_ phase of sperm cell cycle (3.26% ± 1.64%: *P* = 0.042) and G_2_/M (15.11% ± 1.41%: *P* = 0.022) as compared to the control group (4.12% ± 0.58%) and G_2_/M (18.84% ± 3.05%), respectively [34]. An increased level of apoptotic sperm was detected after exposure to 2.45 GHz (14.30% ± 1.92%) and mobile phone (13.15% ± 1.25%) as compared with sham exposed group (7.43% ± 1.30%) and (5.93% ± 1.64%), respectively [9, 31].

Spermatogenesis is an active proliferative process consisting of two phases: the mitotic and meiotic phase. The cell cycle is regulated by a control system formed by molecules that trigger and coordinate key events. These molecules act primarily at two important check points in the cell cycle, G0 to G1, and G2 to M [16]. Initiation of the M-phase in the sperm cell cycle requires a protein kinase complex consisting of a catalytic sub-unit [78, 79] and regulatory sub-unit. Assessment of the catalytic activity of a specific protein kinase plays an important role in elucidating signal transduction pathways, which may affect cell behavior.

Kesari et al. have investigated a significant (*P* = 0.003) decrease in the level of sperm PKC activity after mobile phone exposure (2876 ± 617.9 P^32^ counts/mg protein) as compared to the control group (3013 ± 520.67 P^32^ counts /mg protein, where P^32^ is radioactive phosphorus-32 labeled ATP) [9]. Several other studies also reported a decline in sperm motility together with a decrease in PKC activity [80, 81]. This could mediate the cellular response to extra-cellular stimuli involved in proliferation, apoptosis, decreased sperm count, and exocytotic discharge in a number of non-neuronal cells i.e. sperm [31, 82]. Kesari et al. have reported a significant decline (*P* = 0.006) in sperm histone kinase activity in a microwave-exposed group (3659.08 ± 1399.40 P^32^ counts/mg protein) as compared to the sham exposed one (5374.91 ± 1366.91 P^32^ counts/mg protein) [38]. Decrease in histone H1 kinase activity just before the entry of differentiating cells into the M-phase, suggesting an universal role of Cdc2/Cdk2 (cell division cycle/cyclin-dependent kinase) kinase to regulate the G2/M transition [34]. Kumar et al. [30] and Kesari et al. [9] demonstrated that depletion in the activity of both histone kinase and protein kinase may serve as a measure of microwave EMF’s ability to affect spermatogenesis and sperm cell cycle. Kumar et al. has also investigated a significantly increased (*P* < 0.001) level of sperm creatine kinase in the microwave-exposed group (0.24 ± 0.10 IU/10^8^ spermatozoa) compared to the sham group (0.04 ± 0.03 IU/10^8^ spermatozoa) [29].

In spermatozoa, creatine kinase is localized in the mitochondria of the midpiece region [83]. Creatine phosphate serves as a donor for the re-phosphorylation of adenosine diphosphate (ADP) into ATP, which supports flagellar dynein/adenosine triphosphate and sperm quality [84]. Since differences in the creatine kinase activity reflect differences in sperm ATP concentrations and ATP/ADP ratios [84], it can be suggested that protein kinase C, histone kinase and creatine kinase play an important role in cell metabolism and spermatogenesis and any changes in sperm kinases due to RF-EMF or other factors may lead to infertility.

#### RF EMF exposure affects hormonal changes

Microwave exposure disrupts the seminiferous tubules and reduces the Leydig cell population and therefore the serum testosterone concentration in rats. Leydig cells secrete testosterone, where luteinizing hormone (LH) stimulates Leydig cells to produce testosterone and maintains their function. Testosterone is responsible for feedback control of the LH secretion at both the hypothalamus and pituitary. This pituitary hormone promotes the secretion of testosterone by the Leydig cells, which are the interstitial cells situated between the seminiferous tubules [85]. Leydig cells are among the most susceptible cells to EMW and injury to these cells may affect spermatogenesis [86]. Kumar et al. have reported a decline in the level of testosterone after 10 GHz of microwave exposure, where significant differences in exposed animals (1.4 ± 0.8 ng/ml) were found by comparing to the sham-exposed one (4.1 ± 1.4 ng/ml) [63].

Several studies reported that testosterone is essential for spermatogenesis, formation of spermatozoa, and maintenance of structural morphology and physiology of seminiferous tubules [87, 88]. Therefore, any changes in the level of testosterone will have detrimental effects on male fertility. Meo et al. reported that radiations may affect the state of polarization of the cellular membranes [33]. This may be responsible for distinct changes in testosterone synthesis and secretion. Since changes in serum testosterone levels may be associated with a possible effect on pineal melatonin secretion, mobile phones may cause a reduced melatonin production, which is reported in several studies [27, 89, 90]. Melatonin is an important factor in testosterone secretion because it exerts an antigonadotrophic effect mainly at the level of the hypothalamus and pituitary [91–93].

**Fig. 2:**
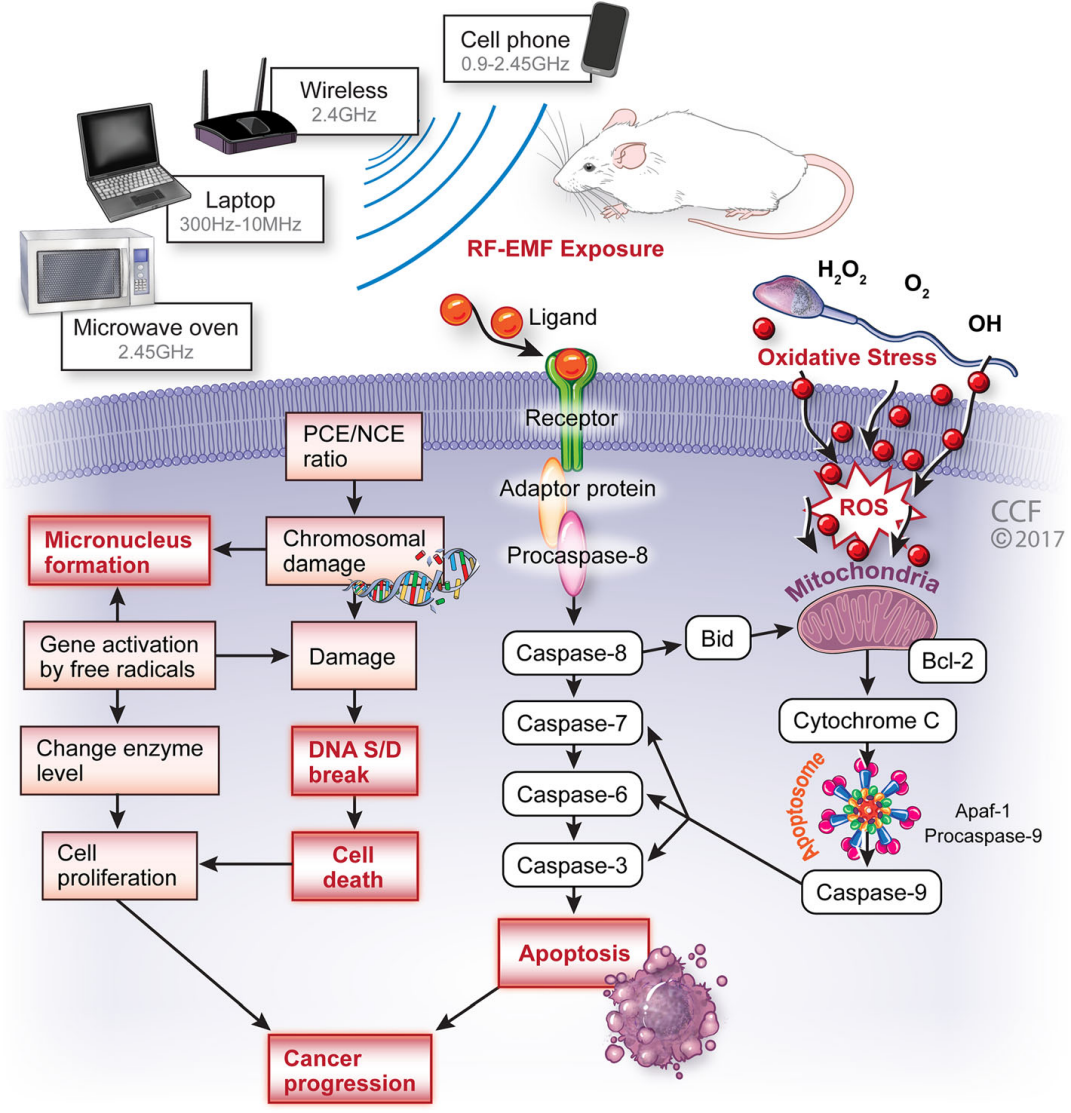
An overview on the effects of RF EMF exposure, emitting from various sources (cell phone, microwave oven, Wi-Fi, Laptop) on genotoxic parameters. The proposed mechanism suggesting radiation-induced oxidative damage may increase DNA damage, micronuclei formation and leading cancer progression. This has been linked to distorted sperm head and mitochondrial sheath in sperm tail which leads to apoptosis and finally cancer progression

RF-EMF exposure and Genotoxicity: Many in vitro and in vivo studies showed that EMF induced genotoxic single- and double-strand DNA breaks, micronucleus formation, chromosomal abbreviations, changes in gene expression, cell proliferation and apoptosis [25, 26, 94–97]. Such changes are responsible for genomic instability and promote tumorigenic effect in cells. We explore the genotoxic effect of RF EMF on sperm parameters and possible infertility outcome as discussed below and which is also represented in Figure 2.

#### DNA damage

The majority of infertile men present with DNA damage [98–100]. Apart from several other lifestyle factors, cell phone use has been identified to induce sperm DNA damages [26] as a result of an overproduction of reactive oxygen species (ROS) in men continuously using mobile phones. This may lead to the development of different pathologies including tumors, and problems in the spermatogenesis [25, 67].

Carrying the cell phone in the trouser pocket impairs the sperm quality. Kumar et al. has reported DNA strand break in sperm cells after exposure of the testes (the antenna position of 3G cell phone kept near rat testis) for 2 h/day for 60 days in this mode [26]. Using the Comet assay, the authors reported significant (*p* < 0.05) increases in sperm DNA tail length (138.03 ± 57.84 μm) and DNA tail moment (34.59 ± 45.02%) in the exposed group as compared to the control (39.96 ± 36.51 μm and 2.75 ± 3.08%), respectively. Kumar et al. has also reported DNA damage when animals were exposed 2 h/day for 45 days to 10 GHz of microwaves exposure [63]. The authors reported a significant (p < 0.05) increase in tail intensity (15.1 ± 13.1%), tail length (154.4 ± 49.4 μm) and tail moment (21.6 ± 14.7%) in the exposed group compared to the control group, where tail intensity (1.5 ± 2.01%), tail length (56.6 ± 14.2 μm) and tail movement (4.0 ± 0.5%) were obtained. The parameters like tail length is the distance of DNA migration from the body of nuclear core; tail moment is the product of the tail length and fraction of total DNA in the tail and tail intensity represents the number of relaxed/ broken pieces of DNA in the tail. It is interesting to note that with the duration of the exposure and an increasing power density (emitted radiation during exposure) the magnitude of the effect also increased.

Recently, Meena et al. have reported a significant increase in sperm DNA damage after whole body microwave exposure at 2.45 GHz for 2 h/day for 45 days by measuring DNA tail length and tail movement by using the comet assay [25]. RF-EMF of 2.45 GHz exposure caused rearrangement of DNA segments and breakage of DNA in the testes [101]. Therefore, any changes at DNA level in sperm or any other cell type may have mutagenic or tumorigenic effects.

Several other pilot studies (in vitro) on the effect of 2.45 GHz RFR on human ejaculated semen found changes in sperm motility and DNA fragmentation [8, 102]. Studies using RF-EMF of 900 MHz and 1.7 GHz revealed induced DNA breakage in cauda epididymal spermatozoa and embryonic stem cells in mice [3, 103]. Since the male germ cell is very compact and rigid in nature, DNA damage due to EMF is significant. However, a short-term effect of RF exposure is not strong and effective enough to cause any genomic level of changes because this damage may be the result of cumulative effects of repeated exposure [16]. Yet, it is also suggested that oxidative stress plays a key role in the underlying mechanism of sperm DNA fragmentation.

#### Micronuclei, chromosomal damages and genomic instability

Micronuclei (MN) is a well-known biomarker of genotoxic events where an induced MN formation led to cell death, genomic instability, or cancer development [104]. Ionizing radiation is also a well-known inducer of genomic instability [3]. Adiga et al. reported that the exposure to ionizing radiation in mice could cause sperm DNA fragmentation and lead to transgenerational genomic instability in the offspring [105]. Radiation induced genomic instability (IGI) can be defined as delayed de novo appearance of genetic alterations after multiple cell generations. Micronuclei have been used to measure radiation-induced chromosomal damage in bone marrow and peripheral blood erythrocytes in rats [63].

Recently, Kesari et al. reported a significant increase in polychromatic erythrocyte (PCE) in a 3G mobile phone-exposed group (132.66 ± 8.62 micro-nucleated PCE/1000 erythrocytes) as compared to the sham exposed (15 ± 3.56 micro-nucleated PCE/1000 erythrocytes, *P* < 0.002) [96]. Similarly, a flowcytometric analysis showed that increased micronuclei formation with the ratio of PCE/NCE (normochromatic erythrocyte) after exposure to 3G mobile phone (0.24 ± 0.02 micro-nucleated PCE/1000 erythrocytes) was significantly lower as compared with the sham-exposed group (0.56 ± 0.05 micro-nucleated PCE/1000 erythrocytes; *P* < 0.001). Kumar et al. have also reported a significant (*P* < 0.0004) increase by 52.75% in micronuclei formation in blood samples after 10 GHz microwaves exposure compared to the control [63]. The measurement of micronuclei formation has been proposed as a reliable method for measuring genotoxic or cytotoxic damages “in vivo” [106].

The basic phenomenon of micronuclei formation is that during red blood cells (RBC) formation, erythroblasts expel their nucleus and damage the chromosomes in the cytoplasm of young erythrocyte (in the form of micronuclei). Due to their small size, the radiofrequency-induced MN are likely to arise via a clastogenic effect [38, 107]. Micronuclei formation due to EMF is responsible for induced genomic instability [108]. Recent in vitro studies using neuronal cell lines suggest that exposure to ELF MFs may induce genomic instability after several generations [108, 109]. Thus far, no studies have reported genomic instability after short-term exposure to RF-EMF. Therefore, it is too early to conclude that any changes due to RF-EMF with decreased sperm count, motility, chromosomal or DNA damage and micronuclei formation may lead to the genomic instability. Nevertheless, such effect after long term RF-EMF exposure detection might be serious concern.

#### Microtubule and mitochondrial function

The physiology of sperm is an important factor in the fertility pattern, where microtubules participates and play a crucial role in cell division, intra-cellular transport, maintenance of cell polarity and motility. Any severe changes in the sperm structure (head: nucleus, acrosome; mid piece: mitochondria; flagellum) leads to decreased sperm count, decreased motility and finally infertility. The manchette and axoneme have a very important role as being part of the formation or in development of sperm head and tail [110], of which the main constituents of the latter are microtubules [111]. Any alteration in the ultrastructure of these microtubule-based structures may cause abnormalities in the sperm tail and alter its morphology causing severe alterations in its motility and are thus associated with infertility [112].

Kesari and Behari investigated an alteration in microtubule arrangement after exposure to mobile phone radiation [28]. Results observed under transmission electron microscopy of spermatozoa from RF-exposed rats showed significant changes in the midpiece region, microtubules of axoneme, and outer dense fibers of mitochondria and membranes. These authors also reported that the sagittal section of sperm nucleus with the acrosome shows a distortion (diffusion) from membrane head. The axoneme is the inner core structure of the cilia and flagella and is composed of a typical 9 + 2 pattern, two central and nine peripheral microtubules doublets. It originates from the distal centriole of the round spermatid centrosome [113]. For the generation of motility, the flagella and their microtubule assembly need a source of energy, where ATP hydrolysis provides the chemical energy required for production of kinetic energy, i.e. flagellar movement.

ATP is produced by the mitochondria present in the anterior section of sperm tail called the mid piece. Excess exposure of sperm to mobile phone RF-EMF causes a disruption of sperm mitochondria and resulted in production of high levels of ROS [67], which in turn are responsible for the decrease in sperm motility and the distortion of the acrosome possibly leading to an inability to penetrate oocytes, causing in infertility [28]. Figure 2 represents the possible mechanism of RF-EMF induced oxidative damage in mitochondria of sperm tail. However, several researchers reported that due to an excessive mitochondrial ROS production, the sperm cells’ limited endogenous antioxidant defenses are rapidly overwhelmed, which in turn may induce oxidative damage leading to peroxidation of the sperm acrosomal membrane and diminished acrosin activity [114, 115].

### RF induced oxidative stress and ROS formation

The link between RF-EMF exposure and possible health effects are associated with the production of reactive oxygen species (ROS) and as a result of that increased oxidative stress. Oxidative stress is a condition in which the natural balance between oxidants and antioxidants is derailed towards an excessive amount of oxidants in relation to the antioxidants. This condition leads to biological damage of cells, tissues and organs [116]. De Iullis et al. reported that oxidative stress might be the main factor causing an elevation in sperm chromatin/ DNA damage [67]. However, exposure to cell phone radiation may induce oxidative stress leading to enhanced lipid peroxidation and changes in the antioxidant activities in the body [117]. Although, seminal plasma has a high capacity of endogenous antioxidants in order to protect spermatozoa from oxidative damage [118, 119], cell phone exposure leads to the induction of oxidative stress through the generation of ROS in the sperm plasma membrane by activation of NADH oxidase and similarly the activation of leukocytes.

Spermatozoa are particularly vulnerable to RF-induced oxidative stress. Small changes in the ROS level may play an important role in sperm capacitation, the acrosome reaction, and binding to the oocyte [120]. Kesari et al. observed significantly (*P* = 0.035) increased ROS levels as expressed as mg H_2_O_2_/l (58.25 ± 10.36 mg/l) in semen of rats that were exposed to mobile phone radiation. In the control group, the ROS levels were 41.78 ± 12.93 mg/l [38]. Kumar et al. has also reported a significant increase in seminal ROS level after 10 GHz of microwave exposure [121].

Many researchers have reported that elevated levels of ROS are cytotoxic, and may results in a loss of sperm motility, count and vitality [122–125]. Since sperm motility is directly associated with mitochondrial dysfunction, defects in sperm mitochondrial ultrastructure could be associated with decreased sperm motility in humans [126, 127].

The existence of deteriorated spermatozoa in the semen significantly increases the production of ROS and leads to mitochondrial dysfunction [128]. Since mitochondria in spermatozoa constantly supply the energy for sperm motility, any metabolic disruption in the electrons transport chain can increase the mitochondrial ROS production significantly, thus affecting sperm motility [129, 130]. Moreover, an increased mitochondrial ROS production leads to DNA fragmentation, decreased sperm motility and viability after mobile phone exposure [67]. Hence, it is important to protect the cells from free radical attacks by scavenging these highly reactive molecules with antioxidants.

Infertile men have significantly increased seminal ROS levels as well as a reduction in the antioxidant capacity compared with fertile controls [18, 131–135]. The formation of ROS may affect several enzymes such as superoxide dismutase (SOD), catalase (CAT) or glutathione peroxidase (GPx), which are found in seminal fluid and protect spermatozoa against the assault of ROS. Kesari and Behari [28] and Kesari et al. [38] have reported a decrease in glutathione and superoxide production after RF-EMF exposure at different frequency and power levels where the decreased glutathione level during sperm production correlated with disruption in the membrane integrity of spermatozoa as consequence of induced oxidative stress.

### RF-EMF exposure affects the reproductive endocrine system

RF-EMF exposure may not only disrupt brain functions which in turn may lead to negative effects on the reproductive endocrine system as the central nervous system (CNS), particularly the limbic system and the hypothalamus, but also play an important role in controlling testicular hormones through neuro-endocrine feedback mechanisms via gonadotropin releasing hormone (GnRH) stimulating follicle-stimulating hormone (FSH) and LH as key hormones released from the pituitary gland. RF-EMF exposure can affect the release of adrenocorticotropic hormone, growth hormone, thyroid stimulating hormone, FSH, and LH in the pituitary [136]. Therefore, any decrease in the level of FSH may negatively affect spermatogenesis. On the other hand, LH stimulates Leydig cells to produce testosterone; therefore a decrease in the level of the testosterone may affect sexual differentiation in the fetus and spermatogenesis in the adult. FSH stimulates the Sertoli cells, thereby activates the seminiferous tubules, resulting in the production of sperm as well as the conversion of testosterone to estradiol [137]. Researchers reported that EMF is also responsible for the decrease of melatonin levels in the brain pineal gland [27, 138]. Oktem et al. also found decreased melatonin concentrations due to microwave radiation-induced increased oxidative stress [139]. Melatonin exerts an antigonadotrophic effect mainly at the level of the hypothalamus and pituitary [91, 92] and decreases the testosterone secretion in Leydig cells with relevantly decreased testicular size and insufficient testosterone production [92]. Melatonin regulates the pulse of LH secretion in the hypothalamus, influencing gonadotropin FSH and LH release. Eventually, this can alter the production of gonadal sex steroids, resulting in changes in the reproductive cycle [140, 141].

A disrupted endocrine system may pose a great risk during prenatal and early postnatal development especially the brain development phase as reported by Sharma et al. [45]. These authors exposed pregnant female mice to 10 GHz microwave radiation and found that the radiation affected the neonatal brain much higher after exposure at 0.25 days of gestation as compared to 11.25 days, indicating the sensitivity of the brain to high frequency radiation during the early developmental phase. More interestingly, Kesari and Behari have reported that progeny from RF-exposed (2 h/day for 45 days) rats showed significant decreases in number and weight as compared with control animals [28].

### Consequences of radiotherapy on male fertility

Vakalopoulos et al. [142] reported that cancer treatments, including surgery, radiotherapy and chemotherapy, could have a transitory as well as a permanent detrimental impact on male fertility. However, in patients with testicular cancer, radiotherapy has been found more deleterious to fertility than chemotherapy [143], an observation which has not been confirmed by some other authors [144, 145]. The doses applied for radiotherapy range from 3000 to 7000 cGy and are found to have mutagenic, teratogenic and embryotoxic effects [146, 147]. The constant production of sperm in the germinal epithelium renders the testes as a prime target for radiotherapy, which affects the gonads by damaging sperm production, thus leading to infertility [148]. The extent of the damage caused by radiation depends on the dosage and exposure methods (radiotherapy alone or in combination with other treatment methods). Since spermatogonia are mitotically active, the dividing spermatogonia are most vulnerable to radiation treatment [149]. The estimated dosage of radiation causing adverse effects and a reduction in the number of spermatogonia and daughter cells has been reported as between 0.1–1.2 Gy, while irreversible damage occurs at 4 Gy and a decrease in sperm count is obvious at 4–6 Gy [150]. Damage to Leydig cells is generally associated with infertility [151]. However, these cells are more resistant to radiation-induced injury [152].

During the first 50–60 days after moderate levels of irradiation (1.5–2 Gy dose), the sperm count is reduced up to 50%, which may even lead to azoospermia after moderate-to-high dose irradiation [153, 154, 148]. Post-radiation sperm cell damage is most severe 4 to 6 months after completion of a radiotherapy leading to azoospermia [155]. Whereas, in some men, low sperm counts, decreased motility, and increased rates of chromosomal abnormalities were observed after irradiation [156, 157]. A single dose of radiation administered in multiple treatments, lowers the semen volume and sperm count, which may depend on the dose applied. The recovery period for normal semen volume and sperm count could be 9–18 months if the radiation dose is below 1 Gy, about 30 months after 2–3 Gy exposure and 5 or more years for a dose of 4–6 Gy [148, 158, 159]. In general, the extent of the damage and thus the degree of fertility impairment depends on the radiation dosage. Essentially, any electromagnetic radiation including those deriving from cell phone, cell phone towers, laptop, microwave oven etc. may lead to detrimental effects on fertility. However, the harmful effects of electromagnetic radiation have not been proven in human studies due to inherent limitations associated with carrying out human studies. Therefore, more innovative basic research is needed to decipher and prove the harmful effects of electromagnetic radiation on male fertility.

### Protective measures of RF-EMF exposure

The role of antioxidants in cell protection against RF-EMF-induced oxidative stress has been discussed earlier. Melatonin, N-acetyl-cysteine, and green tea or medicinal plant leaf extracts have antioxidative properties to protect the cells from any damage. The antioxidative properties of melatonin were reported first by Ianas et al. [160] and subsequently by others [25, 161–163]. Melatonin reduces oxidative stress and protects membrane lipids, cytosolic proteins, nuclear and mitochondrial DNA from oxidative damage [164]. In addition, it acts as potent antioxidant to detoxify ROS and stimulates antioxidative enzymes [139, 165]. Moreover, melatonin not only protects the cells from EMF-induced oxidative damage, but also prevents a decline in the mitochondrial membrane potential, which may trigger mitochondrial transition pore opening and triggering the apoptotic cascade [166–168]. A study by Meena et al. reported a protective role of melatonin against microwave radiations [25]. Authors exposed the animals for 2 h per day for 45 days. Melatonin was found to provide protection from oxidative damage as indicated by significant decreases (*p* < 0.001) in the levels of malondialdehyde and ROS (*p* < 0.01). Melatonin treatment also reversed the effects of EMF for sperm count, testosterone level and DNA fragmentation [25].

Consumption of green tea (*Camellia sinensis*), a rich source of polyphenolic compounds, shows promising antioxidant effects [55, 56] as these compounds have anti-inflammatory and anti-oxidative properties. It can also protect from many kinds of diseases due to its anti-proliferative, anti-mutagenic, anti-bacterial, and chemo-preventive properties [169–172]. Reportedly, RF-EMF induces oxidative stress and promotes sperm dysfunctions [10, 25, 26]. However, the consumption of green tea has been found to improve the quality of male and female gametes [173]. These polyphenols are potentially strong to inhibit ROS formation and have a preventative role against RF radiations. Daily consumption of green tea extract could protect the cardiovascular system [174] and lower blood glucose and cholesterol levels [175]. Recently, Roychoudhury et al. suggested that the supplementation of green tea in males could significantly improve sperm parameters by reducing oxidative stress [173]. Several other studies also support that consumption of green tea may alleviate oxidative stress and maintain reproductive health [176, 177]. Kim and Rhee reported that supplementation with green tea catechins significantly reduced the oxidative damage in the microwave exposed group [178]. Zahedifar and Baharara have also reported that green tea has an inhibitory effect and it decreases the average number of micronuclei in cell phone exposed mice [179].

## Conclusion

Studies reveal that the exposure to cell phones, microwave ovens, laptops, or Wi-Fi produces deleterious effects on the testes, which may affect sperm count, morphology, motility, an increased DNA damage, causing micronuclei formation and genomic instability, as well as disruptions in protein kinases, hormones and antioxidative enzymes. Such effects were found to be responsible for infertility due to an over-production of ROS in exposed cells. Studies suggest that the abnormalities reported due to RF-EMF-exposure depend on physical parameters such as duration of the exposure, distance to the source of radiation, power density, and depth of the penetration. Unfortunately, current studies are unable to suggest a true mechanism of how RF-EMF radiation affects the male reproductive system. Therefore, more studies are necessary to provide better evidence of RF-EMF radiations emitted from cell phones, microwaves, Wi-Fi and Wi-Fi-connected laptops, which can be provided by in vitro and in vivo studies in combination with physical bio-modeling. Moreover, very limited research is available on protective measures, which actually worsens the problem as the electro-smog pollution is constantly increasing and one could then expect even more health problems including increased rates of male infertility due to such kind of radiation. On the other hand, possible protective effects of various antioxidants should be elucidated. Yet, this would only address the problem at symptomatic level.
